# Comparison of Patient-Reported Outcomes after Local Flap Coverage versus Amputation for Complex Lower Extremity Trauma

**DOI:** 10.1055/s-0044-1791741

**Published:** 2024-10-24

**Authors:** Neel Bhagat, Connor Drake, Steven Dawson, Scott N. Loewenstein, Kevin R. Knox, Joshua M. Adkinson, Aladdin H. Hassanein, Ravinder Bamba

**Affiliations:** 1 Indiana University School of Medicine, Indianapolis, Indiana, United States; 2 Division of Plastic Surgery, Indiana University School of Medicine, Indianapolis, Indiana, United States; 3 Cimisurgical, Maywood, New Jersey, United States

**Keywords:** limb salvage, local flap, lower extremity, quality of life, patient-reported outcomes

## Abstract

**Background** There is a paucity of patient-reported outcomes (PROs) data in lower extremity salvage. Limb salvage can often be achieved with the use of local muscle flaps or fasciocutaneous flaps. The purpose of this study was to compare PROs of patients who underwent lower extremity salvage using local fasciocutaneous flaps or muscle flaps to lower extremity amputation.

**Materials and Methods** The outcomes of 61 patients that underwent lower extremity local flap reconstruction ( *n* = 33) or amputation ( *n* = 28) between 2014 and 2020 were recorded. Chart reviews were performed to collect perioperative data. Patients were contacted via telephone for participation in the survey portion of our study. PROs were recorded utilizing both the Lower Extremity Functional Scale (LEFS) and the 36-Item Short-Form Health Survey (SF-36).

**Results** Surveys were completed by 61 patients (response rate 59.2%). The mean time of survey after flap reconstruction or amputation was 2.7 ± 1.4 years. Recent trauma (within 90 days) was the most common indication for local flap coverage ( *n* = 23). LEFS score and SF-36 physical functioning scores were significantly lower in patients who underwent muscle flaps compared with fasciocutaneous flaps ( *p* = 0.021 and *p* = 0.022). Muscle flap patients had similar LEFS and SF-36 scores to amputation patients, while fasciocutaneous flap patients had significantly higher LEFS ( *p* = 0.01), SF-36 physical functioning ( *p* = 0.031), physical role functioning ( *p* = 0.031), and emotional role functioning ( *p* = 0.047) scores than amputation patients.

**Conclusion** Patients who underwent local fasciocutaneous flaps for limb salvage reported higher PRO scores than those undergoing amputation, while patients undergoing muscle flaps reported outcomes similar to those undergoing amputation. PROs for muscle flap patients were significantly lower than those of fasciocutaneous flap patients. These data suggest that while fasciocutaneous and muscle flaps are both useful limb salvage procedures, fasciocutaneous flaps may confer advantages that result in improved patient-perceived outcomes. Further study is needed to better characterize outcomes in limb salvage.

## Introduction

Management of complex lower extremity injuries is a significant challenge. The decision to pursue lower extremity salvage versus amputation is complex and multifactorial. Given the common mechanisms of injury for lower extremity trauma, patients typically have coexisting injuries requiring the collaboration of multiple surgical teams. 1 In addition, the short- and long-term consequences of such injuries are often significant for the patient, resulting in a decrease in quality of life (QoL). 2 Despite the impact, QoL studies after lower extremity reconstruction are lacking. 3

Over the past several years, lower extremity reconstruction has been more frequently utilized as an alternative to amputation. 4 5 6 Local options for lower extremity reconstruction include fasciocutaneous and muscle flaps. Fasciocutaneous flaps (e.g., reverse sural, propeller flaps) provide coverage, while sparing muscle. Muscle flaps, such as gastrocnemius or soleus, are considered workhorse reconstructive options, especially for proximal and middle-third lower extremity wounds, respectively. 4 5 6 A potential advantage of local flaps over a free flap is reduced donor site morbidity. However, the use of pedicled muscle flaps may result in weakness from the donor site. This has the potential to affect QoL in limb salvage patients.

With increasing use of free and local flap reconstruction, treatment options for lower extremity trauma have expanded. Studies have shown that limb salvage is psychologically more favorable for patients, compared with amputation. 7 Additionally, tools like Mangled Extremity Severity Score have been developed to evaluate the extent of lower extremity injury and utilized in an attempt to determine whether limb salvage is possible. 8 9 10 11 However, literature on the patient-reported outcomes (PROs) after reconstruction remains sparse. The Lower Extremity Functional Scale (LEFS) is a validated PROs tool that has been used extensively for evaluation after lower extremity trauma and orthopaedic injury. 12 13 The purpose of this study was to compare PROs for those patients who underwent local flaps for lower extremity reconstruction to those who underwent below-knee amputation.

## Materials and Methods

### Study Design

This retrospective study was approved by the Institutional Review Board of Indiana University Medical Center. Inclusion criteria were age > 18 years with lower extremity wounds that required flap reconstruction or amputation between 2014 and 2020. Patients with lower extremity wounds that were managed with primary closure, free tissue transfer, and split-thickness skin grafts without flaps were excluded.

### Patient Recruitment

Eligible patients were contacted via telephone to obtain consent and to administer surveys. Patients were informed that the survey was voluntary and that there was no compensation for the study. Patients were included if they agreed to participate and were able to complete the survey.

### Surveys

Outcomes were assessed using the 36-Item Short-Form (SF-36) health survey 14 15 16 and LEFS survey. 12 We chose SF-36 and LEFS for a multitude of reasons. The SF-36 is a commonly used and well-accepted PRO measure. It provides a comprehensive assessment of a patient's health status across multiple dimensions. LEFS is a specific, reliable, and sensitive measure of lower extremity function. It has been validated in various populations, ensuring that it is a reliable and accurate measure of lower extremity function. Since both of these scales are widely used, a large amount of comparative data is available allowing for benchmarking against other populations or studies. The SF-36 is a self-reported health-based QoL questionnaire, consisting of eight scale scores. The scores range from 0 to 100, with higher scores being indicative of better self-reported health. 14 15 16 The LEFS is a 20-question survey that assesses the functionality of lower extremities. Scale scores range from 0 to 80, with higher scores being indicative of better self-reported lower extremity functionality. 12 Normative data has previously been reported in the literature for SF-36 and LEFS. 17 18 Our patients were compared with previously reported normative data in addition to being compared with each other to establish the burden of lower extremity injury compared with the general population as well as to different treatment methods in lower extremity trauma. Surveys were performed via telephone in the setting of the coronavirus disease 2019 pandemic restrictions. All surveys were performed by the first, second, and third authors (N.B., C.D., S.D.).

### Data Collection

Patient-related variables were extracted from electronic medical records. Patient demographics, past medical history, amputation and/or reconstruction etiology, perioperative treatment characteristics, short-term outcomes (postoperative complications such as wound dehiscence and infection), and long-term outcomes (success of limb salvage) were collected. Flaps were categorized as either fasciocutaneous flaps (e.g., propeller, reverse sural, adipofascial turnover) or muscle flaps (e.g., soleus, gastrocnemius, hemisoleus turndown, tibialis anterior). We collected both the category of flap (i.e., fasciocutaneous or muscle) as well as the specific type of flap. Long-term flap outcomes, such as reoperation and successful limb salvage, were also recorded.

Study data was collected and managed using Research Electronic Data Capture (REDCap) electronic data capture tools hosted at Indiana University. 19 REDCap is a secure, Web-based application designed to support data capture for research studies, providing (1) an intuitive interface for validated data entry, (2) audit trails for tracking data manipulation and export procedures, (3) automated export procedures for seamless data downloads to common statistical packages, and (4) procedures for importing data from external sources.

### Statistical Analysis

All analyses were performed within SPSS Statistics version 19 (IBM Corporation, Chicago, Illinois, United States). Two-tailed values of *p* < 0.05 were considered significant.

## Results

### Patient Demographics

Between 2014 and 2020, 248 patients underwent local flap and 449 patients underwent lower extremity amputation for lower extremity trauma. All patients were contacted via telephone. After excluding those that were deceased or could not be contacted, there were a total of 103 patients, of which 61 patients agreed to fill out the survey (response rate 59.2%). Mean patient age was 50.0 ± 14.2 years. Male patients ( *n* = 43) comprised 70.5% of the study sample. Additional patient demographics and comorbidities are listed in Table 1 .

**Table 1:** Demographics of 33 patients with lower extremity trauma treated with a local flap and 28 patients with lower extremity amputation

Characteristic	Local flap no. (%)	Amputation no. (%)	*p* -Value
No.	33	28	
**Sex**			
Male	23 (69.7)	20 (71.4)	1.0
Female	10 (30.3)	8 (28.6)	
Mean BMI ± SD, kg/m ^2^	31.7 ± 8.4	32.0 ± 7.2	0.91
Mean age at accident ± SD, y	50.0 ± 13.5	50.0 ± 15.3	1.0
Length of patient stay ± SD, d	10.6 ± 12.4	8.0 ± 6.4	0.31
Smoker status at time of surgery	10 (30.3)	10 (38.5)	0.79
Hyperlipidemia	7 (21.2)	13 (46.4)	0.055
Cardiovascular disease	1 (3.0)	11 (39.3)	0.001
Diabetes mellitus	3 (9.1)	6 (21.4)	0.28
Psychiatric diagnosis	12 (36.4)	11 (39.3)	1.0
Race			
Caucasian	30 (90.9)	25 (89.3)	0.71
Black/African American	1 (3.0)	2 (7.1)	
Hispanic	1 (3.0)	1 (3.6)	
Asian	1 (3.0)	0 (0)	

Abbreviations: BMI, body mass index; SD, standard deviation.

### Injury Details

Respondents had a mean follow-up time of 2.7 ± 1.4 years after the initial trauma or amputation operation. In reconstruction patients ( *n* = 33), indications for wound coverage included recent trauma (69.7%, *n* = 23), infection after recent trauma (15.2%, *n* = 5), and remote trauma (15.2%, *n* = 5). For patients undergoing below-knee amputation, indications for amputation included recent trauma (42.9%, *n* = 12), infection after recent trauma (7.1%, *n* = 2), and remote trauma (50%, *n* = 14) ( Table 2 ).

**Table 2:** Characteristics of lower extremity injury in 33 patients treated with local flap reconstruction and 28 patients treated with amputation

Characteristic	Local flap no. (%)	Amputation no. (%)	*p* -Value
No.	33	28	
Wound in need of coverage			
Recent trauma	23 (69.7)	12 (42.9)	0.04
Infection after trauma (< 90 d)	5 (15.2)	2 (7.1)	
Chronic wound from trauma (> 90 d)	5 (15.2)	14 (50.0)	

### Operative Details

Fasciocutaneous flaps were performed in 51.5% ( *n* = 17) of the survey respondents and muscle flaps were performed in 48.5% ( *n* = 16). Gastrocnemius flaps were the most common muscle flaps (27%, *n* = 10), and reverse sural flaps were the most common fasciocutaneous flaps (37.8%, *n* = 14). Additional operative details for reconstruction patients are listed in Table 3 .

**Table 3:** Flap reconstruction details for patients undergoing lower extremity salvage

Characteristic	No. (%)
No.	33
Flap type	
Muscle flap	16 (48.5)
Gastrocnemius	8 (24.2)
Soleus	5 (15.2)
Hemisoleus turndown	2 (6.1)
Anterior tibial	1 (3)
Fasciocutaneous flap	17 (51.5)
Reverse sural	12 (36.4)
Fasciocutaneous propeller	3 (9.1)
Adipofascial turnover	2 (6.1)

### Postoperative Outcomes

In the flap reconstruction group, 6.1% ( *n* = 2) of patients required additional subsequent local flap coverage. Flap complications included wound dehiscence in 18.2% ( *n* = 6) and infection in 9.1% ( *n* = 3). Other flap complications included partial flap necrosis in 30.3% ( *n* = 10) and total flap necrosis in 3.0% ( *n* = 1) ( Table 4 ). There were no immediate complications in the amputation group.

**Table 4:** Postoperative complications in 33 lower extremity trauma patients treated with local flap reconstruction

Characteristic	No. (%)
No.	33
Flap complications	8 (24.2)
Wound dehiscence	6 (18.2)
Infection	3 (9.1)
Secondary procedures	24 (72.7)
Secondary flap coverage	2 (6.1)
Free flap	0 (0)
Final outcomes	
No flap necrosis	22 (66.7)
Partial flap necrosis	10 (30.3)
Total flap necrosis	1 (3.0)

### Patient-Reported Outcomes

The LEFS mean score of all successful reconstruction patients was 42.1 ± 14.2, and the LEFS mean score of amputation patients was 36.5 ± 14.6 ( *p* = 0.136), both of which are lower than that of the general population (median LEFS in general population is 77). 17 SF-36 scores reported in both groups were lower than previously reported normative data from randomly selected healthy subjects aged 18 to 64. 18 LEFS and SF-36 physical functioning scores were significantly lower ( *p* = 0.021, *p* = 0.022) in patients who underwent muscle flaps compared with those who underwent fasciocutaneous flaps. There were no statistically significant differences in SF-36 or LEFS scores between amputation and muscle flap patients. However, amputation patients had significantly lower SF-36 physical function, physical role functioning, emotional role functioning, and LEFS scores than patients who received fasciocutaneous flaps ( *p* = 0.027, *p* = 0.031, *p* = 0.047, *p* = 0.010). Additional survey data are listed in Tables 5 6 7 .

**Table 5:** Survey results of 33 patients treated with local flaps for lower extremity trauma

Characteristic	Mean ( *n* = 33)	Muscle flap ( *n* = 16)	Fasciocutaneous flap ( *n* = 17)	*p-Value*
Time between trauma and survey ± SD, y SF-36 (out of 100)	3.1 ± 1.5	3.3 ± 1.7	2.9 ± 1.3	
Physical functioning	43.03 ± 21.54	34.38 ± 17.78	51.18 ± 22.05	0.022
Physical role functioning	38.64 ± 33.71	28.13 ± 31.46	48.53 ± 33.62	0.08
Emotional role functioning	70.73 ± 41.48	66.69 ± 40.41	74.53 ± 43.35	0.60
Energy fatigue	44.09 ± 19.38	43.75 ± 22.10	44.41 ± 17.13	0.92
Emotional well-being	71.88 ± 23.12	70.75 ± 24.90	72.94 ± 22.02	0.79
Social functioning	75.94 ± 28.90	71.31 ± 30.46	80.29 ± 27.55	0.38
Pain	50.42 ± 28.10	49.44 ± 30.65	51.35 ± 26.40	0.85
General health	58.94 ± 26.45	55.00 ± 28.46	62.65 ± 24.69	0.42
Health change	66.67 ± 24.74	59.38 ± 25.62	73.53 ± 22.48	0.10
LEFS (out of 80)	42.09 ± 14.18	36.31 ± 13.91	47.53 ± 12.50	0.021

Abbreviations: LEFS, Lower Extremity Functional Scale; SD, standard deviation; SF-36, 36-Item Short-Form Health Survey.

**Table 6:** Survey results of 16 patients treated with local muscle flaps, and 28 patients treated with amputation, for lower extremity trauma

Characteristic	Muscle flap ( *n* = 16)	Amputation ( *n* = 28)	*p* -Value
SF-36 (out of 100)			
Physical functioning	34.38 ± 17.78	33.75 ± 28.44	0.929
Physical role functioning	28.13 ± 31.46	25.00 ± 34.69	0.762
Emotional role functioning	66.69 ± 40.41	46.77 ± 44.44	0.139
Energy fatigue	43.75 ± 22.10	43.57 ± 24.53	0.980
Emotional well-being	70.75 ± 24.90	63.57 ± 27.95	0.385
Social functioning	71.31 ± 30.46	63.93 ± 33.65	0.462
Pain	49.44 ± 30.65	55.59 ± 33.24	0.539
General health	55.00 ± 28.46	57.32 ± 28.27	0.796
Health change	59.38 ± 25.62	59.82 ± 24.85	0.956
LEFS (out of 80)	36.31 ± 13.91	36.50 ± 14.57	0.967

Abbreviations: LEFS, Lower Extremity Functional Scale; SF-36, 36-Item Short-Form Health Survey.

**Table 7:** Survey results of 17 patients treated with local fasciocutaneous flaps, and 28 patients treated with amputation, for lower extremity trauma

Characteristic	Fasciocutaneous flap ( *n* = 17)	Amputation ( *n* = 28)	*p* -Value
SF-36 (out of 100)			
Physical functioning	51.18 ± 22.05	33.75 ± 28.44	0.027
Physical role functioning	48.53 ± 33.62	25.00 ± 34.69	0.031
Emotional role functioning	74.53 ± 43.35	46.77 ± 44.44	0.047
Energy fatigue	44.41 ± 17.13	43.57 ± 24.53	0.893
Emotional well-being	72.94 ± 22.02	63.57 ± 27.95	0.220
Social functioning	80.29 ± 27.55	63.93 ± 33.65	0.084
Pain	51.35 ± 26.40	55.59 ± 33.24	0.639
General health	62.65 ± 24.69	57.32 ± 28.27	0.511
Health change	73.53 ± 22.48	59.82 ± 24.85	0.065
LEFS (out of 80)	47.53 ± 12.50	36.50 ± 14.57	0.010

Abbreviations: LEFS, Lower Extremity Functional Scale; SF-36, 36-Item Short-Form Health Survey.

## Discussion

When deciding between limb salvage and amputation, there are many considerations for patients and surgeons. Two important factors to consider are QoL and lower extremity function. A greater understanding of PROs data after reconstruction or amputation for lower extremity trauma may guide decision-making. Our study demonstrated globally low LEFS and SF-36 scores in patients who experienced lower extremity trauma compared with the general population. 20 Muscle flap patients had no significant difference in PROs than those undergoing amputation. However, fasciocutaneous flap patients had better PROs compared with amputation patients and muscle flap patients.

Wound coverage for leg defects is often dictated by the exact location of the wound. Upper and middle third leg wounds are frequently addressed with local muscle flaps, such as pedicled gastrocnemius or soleus flaps. Lower third leg wounds have traditionally required free tissue transfer. 6 Better understanding of vascular anatomy and the perforasome theory has led to the development of locally designed flaps for the lower extremity. 21 Adipofascial turnover flaps, 22 23 24 propeller flaps, 25 26 and reverse sural artery flaps 27 28 29 30 are some of the most commonly used local flaps for lower leg wounds. With multiple options often available for coverage of lower extremity wounds, form and function may play a larger role in wound coverage planning.

Advantages of local flaps for lower extremity reconstruction include reliable wound coverage without the need for microvascular anastomosis. Additionally, the operative times are shorter, and the donor site morbidity is less than free tissue transfer reconstruction. In a comparative effectiveness analysis, Kozak et al found that local flaps for lower extremity reconstruction have lower length of stays, lower costs, and fewer reoperations. 31 Given these and similar data, local flaps have become a powerful tool in the armamentarium of the reconstructive surgeon.

Whereas adequate coverage is a priority in the immediate setting, there should be consideration of ultimate lower extremity function. There has been an expanding interest in PROs in lower extremity reconstruction. 32 Given the complexity and multisystem nature of lower extremity trauma, generic measures of health are implemented into these studies such as SF-36 14 15 and Sickness Impact Profile. 33 Lower extremity outcome measures include Locomotor Capabilities Index, 34 35 LEFS, 12 and Houghton Scale. 20 In our study, we chose to use the SF-36 and LEFS and found them to be relevant in our patient population.

Previous studies have reported varying outcomes when comparing lower extremity salvage and amputation. Bosse and colleagues showed similar Sickness Impact Profile scores and return to work rates between patients who underwent lower extremity salvage and amputation at 2 years postoperatively. Equivalent functional outcomes were confirmed at 7 years postoperatively in a follow-up study of the same cohort. 36 In contrast, Dagum et al 37 studied 55 patients who experienced severe lower extremity trauma and found worse physical function scores in patients who underwent amputation and that 92% of patients preferred limb salvage over amputation. The variation in outcomes of patients undergoing limb salvage versus amputation is likely the result of lower extremity trauma being a heterogeneous population with varying degrees of injury. Injury grading scales such as the Gustilo classification will not always provide a complete picture of the lower extremity trauma. Additionally, there are multiple patient-specific factors that should be included when considering the decision between amputation and limb salvage. 38

Consistent with previous QoL studies for trauma patients, we found that our patient population, regardless of fasciocutaneous flap, muscle flap, or amputation, had lower SF-36 scores than the general public. 18 39 We utilized scores from the general population for the SF-36 and LEFS derived from previously reported large-scale studies. These studies were selected because they are recognized validation studies of these PRO measures, involving surveys of thousands of patients. The results are indicative of the long-term physical and mental stress lower extremity trauma or loss can have on patients. As expected, our patients also had lower LEFS scores compared with the general public. 17 However, the results of this study indicate that fasciocutaneous flaps may result in better long-term lower extremity function and patient well-being, both in comparison to amputation and muscle flap reconstruction options. Our findings suggest that reconstructive surgeons should consider potentially diminished function after muscle flap reconstruction. In separating local flap reconstruction into muscle and fasciocutaneous, we found a difference in PROs when comparing fasciocutaneous flaps to amputation patients as a control group; however, no difference was noted between amputation and muscle flap reconstruction. These findings suggest that, despite globally low PROs in comparison to a healthy population, there is an important role for lower extremity reconstruction, and choice of reconstructive option can significantly impact patient-perceived outcomes.

Our study has several limitations, including the retrospective nature of the study. Our sample size was limited due to inability to contact many patients. Given the nature of our patient population derived from a Level 1 trauma center, we were not able to reach a significant amount of our patients. As a retrospective analysis, the follow-up times were also variable, which could affect QoL scores. Another limitation for our study was the exclusion of those undergoing free flaps for limb salvage. At our institution, we often employ local flaps such as reverse sural flaps and propeller flaps for wounds that classically would be covered with a free flap. Therefore, we chose to exclude free flaps from the study and to focus on local flaps. Given the potential use of free flaps in large lower extremity wounds, this may have introduced selection bias into our study.

Additionally, patients undergoing amputation in our study had higher incidences of diabetes and cardiovascular disease, which may have impacted decision-making for limb salvage. Our study found higher rates of diabetes in the amputation group compared with the local flap group; however, this difference did not reach statistical significance. We do not believe that a diabetes diagnosis influenced the decision for amputation over a local flap. Nonetheless, given these numbers, diabetes could still be a confounding factor in our study. In a larger study, the difference in diabetes incidence might have been significant. Here was a statistically higher incidence of cardiovascular disease in the amputation group; however, we do not believe this influenced the decision to proceed with amputation. Both local flap surgery and amputation are comparable in terms of cardiovascular stress for the patient. We documented the presence or absence of cardiovascular disease, regardless of severity, and for many patients, it was a preexisting condition. None of the patients had active cardiovascular issues at the time of surgery that would have affected the choice of procedure. These differences in patient comorbidities were another potential source of selection bias and confounding variables. Despite the limitations in our review, we believe that our results are still valid.

## Conclusion

Lower extremity trauma patients have decreased QoL compared with the general population, and face challenges such as chronic pain, mental stress, and decreased lower extremity function. All patients who underwent lower extremity trauma had PROs that were globally lower than previously reported data for healthy populations. Patients undergoing either muscle flap or amputation had similar PROs; however, PROs of patients undergoing fasciocutaneous flap reconstruction were better than those in muscle flap reconstruction and amputation patients. These data suggest that while fasciocutaneous and muscle flaps are both useful limb salvage procedures, fasciocutaneous flaps may confer advantages that result in improved patient-perceived outcomes.
